# Postmitotic Expression of SOD1^**G93A**^ Gene Affects the Identity of Myogenic Cells and Inhibits Myoblasts Differentiation

**DOI:** 10.1155/2015/537853

**Published:** 2015-09-28

**Authors:** Martina Martini, Gabriella Dobrowolny, Michela Aucello, Antonio Musarò

**Affiliations:** ^1^Institute Pasteur-Cenci Bolognetti, DAHFMO-Unit of Histology and Medical Embryology, IIM, Sapienza University of Rome, Via A. Scarpa 16, 00161 Rome, Italy; ^2^Center for Life Nano Science at Sapienza, Istituto Italiano di Tecnologia, Viale Regina Elena 291, 00161 Rome, Italy; ^3^Edith Cowan University, Perth, WA 6027, Australia

## Abstract

To determine the role of mutant SOD1 gene (SOD1^G93A^) on muscle cell differentiation, we derived C2C12 muscle cell lines carrying a stably transfected SOD1^G93A^ gene under the control of a myosin light chain (MLC) promoter-enhancer cassette. Expression of MLC/SOD1^G93A^ in C2C12 cells resulted in dramatic inhibition of myoblast differentiation. Transfected SOD1^G93A^ gene expression in postmitotic skeletal myocytes downregulated the expression of relevant markers of committed and differentiated myoblasts such as MyoD, Myogenin, MRF4, and the muscle specific miRNA expression. The inhibitory effects of SOD1^G93A^ gene on myogenic program perturbed Akt/p70 and MAPK signaling pathways which promote differentiation cascade. Of note, the inhibition of the myogenic program, by transfected SOD1^G93A^ gene expression, impinged also the identity of myogenic cells. Expression of MLC/SOD1^G93A^ in C2C12 myogenic cells promoted a fibro-adipogenic progenitors (FAPs) phenotype, upregulating HDAC4 protein and preventing the myogenic commitment complex BAF60C-SWI/SNF. We thus identified potential molecular mediators of the inhibitory effects of SOD1^G93A^ on myogenic program and disclosed potential signaling, activated by SOD1^G93A^, that affect the identity of the myogenic cell population.

## 1. Introduction

The function of the metalloenzyme SOD1 is to convert superoxide, a toxic by-product of mitochondrial oxidative phosphorylation, to water or hydrogen peroxide. However, alteration in wild type SOD1 expression or mutations in the gene have been held responsible for the activation of catabolic pathways associated with degenerative diseases, including amyotrophic lateral sclerosis (ALS) [1]. ALS is a disorder involving the degeneration of motor neurons, muscle atrophy, and paralysis [1]. In few familiar forms of ALS, mutations in SOD1 gene have been associated with the pathogenesis of the disease [1]. Initially it has been suggested that mutation in SOD1 gene led to a decrease in the protein enzymatic activity (loss of function hypothesis). However, subsequent studies have clarified that mutant SOD1 possesses a neurotoxic property (gain of function hypothesis) responsible for the pathogenic mechanism of the disease [2]. Indeed, the finding that overexpression of mutant SOD1 in transgenic mice recapitulates several clinical features of ALS disease even in the presence of endogenous mouse SOD1 has led to the conclusion that the disease results from a toxic gain of function [3]. Mutations in SOD1 that impair its functions may lead to increased oxidative damage, promoting the activation of apoptotic pathways.

Oxidative stress plays an important role in the physiopathology of tissues. The effects of the reaction oxidative species (ROS) are dose-dependent, and low ROS concentration is necessary to guarantee cellular homeostasis while high ROS dose exerts toxic effects on the cells and may contribute to cellular dysfunction. Indeed, oxidative stress is a hallmark of aging and several chronic diseases such as Alzheimer's disease, Duchenne dystrophy, and ALS [4]. How such an oxidative insult plays a direct role in the disease-related decrease of muscle performance and mass remains largely unknown. In addition, the discrepancy among different studies has further complicated the achievement of a conclusive link between altered balance of ROS generation and altered homeostasis-associated diseases.

In a previous work we demonstrated that muscle specific expression of the mutant isoform of SOD1 gene (SOD1^G93A^) induces muscle atrophy associated with a significant reduction in muscle strength and alterations in the contractile apparatus [5]. We provided evidences that muscle-restricted expression of SOD1^G93A^ gene is sufficient to increase oxidative stress and to induce a reduction in protein synthesis and the activation of proteolytic pathway [6].

It has been demonstrated that lactate-induced oxidative stress delays C2C12 differentiation [7] while treatment of the same cell line with resveratrol, that confers resistance against oxidative stress, promotes myogenesis and hypertrophy [8]. Interestingly, high glucose-induced oxidative stress has been correlated with lipid deposition in muscle derived stem cells leading to their adipogenic differentiation [9].

In this study, we address the role of the toxic effect of mutant SOD1 gene (SOD1^G93A^) on* in vitro* myogenic program and we demonstrate that SOD1^G93A^ expression prevents myoblasts differentiation and retains C2C12 cells in an undifferentiated state that show features common to fibro/adipogenic cells.

## 2. Materials and Methods

### 2.1. Generation of C2C12 MLC/SOD1^G93A^

C2C12 cells were stably transfected with pPURO and pMexMLC/SOD1^G93A^ plasmids (ratio 1 : 10) by using SuperFect Transfection Reagent (Qiagen) according to the manufacturer's instructions, as control C2C12 cells were also transfected with pPURO and pMex empty vector. After 1 day from transfection the medium was replaced with fresh medium containing puromycin 3 *μ*g/mL (Sigma Aldrich). After 48 hours the cells were split 1 : 10 into the selective medium. The medium was changed every 2 days for 8 days. Single clones were picked, plated in 60 mm dishes, and expanded.

### 2.2. Cell Culture

C2C12 and C2C12 MLC/SOD1^G93A^ cells were maintained in Dulbecco's modified Eagle's medium (DMEM) with 4.5 g/L glucose, L-glutamine (Sigma), supplemented with 15% fetal bovine serum (Gibco), 100 U/mL penicillin (Sigma Aldrich), 100 *μ*g/mL streptomycin (Sigma Aldrich), and 2,5 *μ*g/mL of puromycin (Sigma Aldrich) for C2C12 MLC/SOD1^G93A^ cells. To induce differentiation, cells were shifted to differentiation medium (DM), DMEM with 2% horse serum (Gibco). Cells were harvested at D0, D2 (48 h after shift in DM), and D5 (120 h after shift in DM).

### 2.3. Protein Extraction and Western Blot Analysis

C2C12 and C2C12 MLC/SOD1^G93A^ cells grown in 60 mm culture dishes were washed twice with cold phosphate-buffered saline, pelleted, resuspended in 100 uL of modified lysis buffer (Tris-HCl, ph 7.5/20 mM, EDTA/2 mM, EGTA/2 mM, sucrose/250 mM, DTT/5 mM, Triton-X/0.1%, PMSF/1 mM, NaF/10 mM, SOV4/0.2 mM, and cocktail protease inhibitors/1X (Sigma Aldrich)), and processed for western blot analysis. Filters were blotted with antibodies against hSOD1 (Santa Cruz), gp91phox (BD) anti-Perilipin 2 (Lifespan Biosciences), phospho-p42/p44 MAPK (Millipore), p42/p44 MAPK (Cell Signaling) phospho-Akt (Thr 308) (Sigma Aldrich), Akt (Cell Signaling), phospho-P70 (Thr389) (Cell Signaling), p70 (Cell Signaling), and HDAC4 (Cell Signaling). Protein levels of *α*-tubulin were used as control for equal protein loading. Signals were acquired by ChemiDoc-It Imaging System (UVP, LLC) and the analysis was performed using VisionWorks LS Image Acquisition analysis software.

### 2.4. RNA Extraction and Quantitative RT-PCR

Total RNA extraction was performed using TRIzol reagent method (Sigma Aldrich) as described by the manufacturers. MicroRNA (miRNA) was reverse-transcribed using the TaqMan MicroRNA Reverse Transcription Kit (Life Technologies), and mRNA was reverse-transcribed using QuantiTect Reverse Transcription Kit (Qiagen). The reverse-transcription reactions were performed according to the manufacturers' instructions. Quantitative PCR was performed on an ABI PRISM 7500 SDS (Life Technologies), using premade 6-carboxyfluorescein- (FAM-) labeled TaqMan assays for beta actin, Pax7, Pax3 MyoD, Myogenin, MRF4, and Smarcd3 (Life Technologies). FAM-labeled TaqMan MicroRNA Assays for miR1, miR133a, miR206, and U6 snRNA (Applied Biosystems, USA) were performed as described. Quantitative RT-PCR sample values were normalized to the expression of beta-actin or U6 snRNA for mRNA and microRNA, respectively. The relative level for each gene and miRNA was calculated using the 2-DDCt method [10] and reported as mean fold change in gene expression.

### 2.5. Lipid Staining

Lipid accumulation was visualized by Oil Red O staining. Cells were fixed in 4% paraformaldehyde for 1 h. After being washed with ddH2O, cells were treated with 100% propylene glycol for 5 min and stained with a filtered Oil Red O solution (0.5% Oil Red O in propylene glycol) for 8 min at 60°C. The cells were treated with 85% propylene glycol solution for 5 min, washed twice with ddH2O, and mounted with glycerol. All reagents for this staining were from Sigma Aldrich. Samples were visualized using an inverted microscope (Axioskop 2 plus; Carl Zeiss MicroImaging Inc.).

### 2.6. Trichostatin Treatment

C2C12 and C2C12 MLC/SOD1^G93A^ cells were exposed to 100 nm (Sigma Aldrich) Trichostatin A (TSA) in GM (growth medium) for 24 h. TSA was removed and the cells were analyzed for myosin expression (at day 5 in DM) or by cytofluorimetric profile (at day 2 in DM).

### 2.7. Morphometric Analysis

Cells were fixed in 4% paraformaldehyde and incubated overnight at 4°C with primary antibody against MHC (MF-20 Hybridoma Bank); nuclei were visualized using Hoechst staining. Samples were viewed under an inverted microscope (Axioskop 2 plus; Carl Zeiss MicroImaging Inc.). To quantify the differentiation and fusion of control and TSA treated we calculated the differentiation index as the percentage of MHC-positive cells above total nuclei and the fusion index as the average number of nuclei in MHC-positive cells with at least three nuclei above total number of nuclei, respectively. The images were analyzed using ImageJ software.

### 2.8. Flow Cytometry

C2C12 and C2C12 MLC/SOD1^G93A^ were detached from culture with 0.25% trypsin, 2 mM EDTA (Sigma Aldrich) at indicated times. The isolated cells were then filtered through a 40 *μ*m cell strainer (Falcon) and incubated with the following antibodies (10 ng/mL): CD31-PECy7, CD45-eFluor 450 (eBioscience), Sca-1-FITC (Macs), and *α*7 integrin-PE (R&D Systems Inc.). A subsequent incubation with 7-aminoactinomycin D (1/1000; Sigma) was performed to stain nonviable cells. For each analysis, data were collected from 10,000 cells and analyzed on CyAN ADP (Dako) flow cytometer using the Summit 4.3 software (Dako).

### 2.9. Statistical Analysis

Statistical analysis was performed with GraphPad Prism Software. All data are expressed as mean ± SEM. Groups were compared using nonparametric tests (Mann Whitney Rank Sum test) and Student's* t*-test. A *P* value of <0.05 was considered statistically significant.

## 3. Results

### 3.1. Muscle Specific Expression of Mutant SOD1 Gene Prevents Differentiation of C2C12 Cells

**Figure 1:**
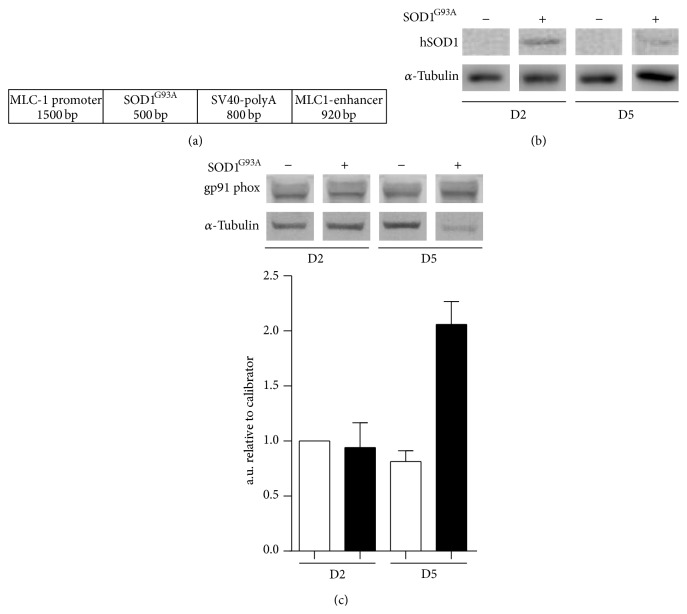
Characterization of C2C12 MLC/SOD1^G93A^. (a) Schematic representation of MLC/SOD1^G93A^ construct. (b) Western blot analysis for mutant human SOD1^G93A^ protein in C2C12 and C2C12 MLC/SOD1^G93A^ at different time points during differentiation. (c) Upper panel shows representative western blot analysis of gp91phox expression in C2C12 and C2C12 MLC/SOD1^G93A^ cells. Lower panel shows the relative densitometric analysis of C2C12 (white bars) and C2C12 MLC/SOD1^G93A^ (black bars). D2 and D5 referred to days of culture in differentiation condition. Data are represented as mean ± SEM.

**Figure 2:**
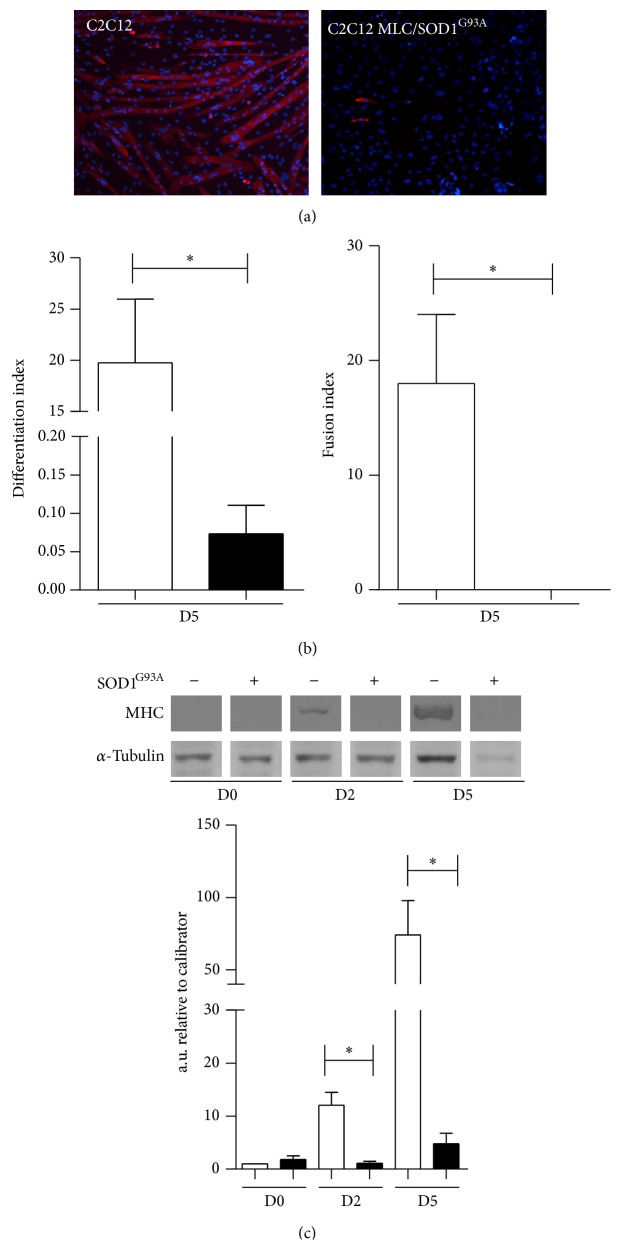
Postmitotic expression of mutant SOD1 gene inhibits C2C12 cells differentiation. (a) Representative immunofluorescence images of C2C12 and C2C12 MLC/SOD1^G93A^ cells stained with anti-myosin heavy chain (MHC) antibody after 5 days in differentiation medium. (b) The histograms represent the differentiation index (left panel) and fusion index (right panel) in control (white bars) and transfected cells (black bars). (c) Upper panel shows representative western blot analysis of MHC expression in C2C12 and C2C12 MLC/SOD1^G93A^ cells. Lower panel shows densitometric analysis for MHC expression in C2C12 (white bars) and C2C12 MLC/SOD1^G93A^ (black bars). D0, D2, and D5 referred to days of culture in differentiation condition. Data are represented as mean ± SEM (^*^ *P* < 0.05).

To investigate the role of mutant SOD1^G93A^ gene in myoblast differentiation we stably transfected the C2C12 cells line with the MLC/SOD1^G93A^ expression cassette (Figure 1(a)) that allows the expression of the mutated isoform of SOD1 gene under the control of the myosin light chain promoter [11]. As expected, SOD1^G93A^ transgene expression was accumulated in C2C12 MLC/SOD1^G93A^ cells induced to differentiate (Figure 1(b)). The postmitotic expression of SOD1^G93A^ transgene induced the accumulation of Gp91 protein, a marker of mitochondria oxidative damage (Figure 1(c)) that might mediate the toxic properties of mutant SOD1 on muscle differentiation and homeostasis. To explore if SOD1^G93A^ transgene expression directly interferes with muscle differentiation, we stimulated differentiation by shifting the cells from growth to the differentiation medium (DM) (Figure 2(a)). Morphological and immunofluorescence analysis revealed a dramatic inhibition of muscle differentiation in C2C12 MLC/SOD1^G93A^ cells, with significant reduction in the number and size of the myosin positive cells, compared with control C2C12 myotubes (Figures 2(a)-2(b)). Morphometric analysis revealed a complete inhibition of fusion index, quantified as the percentage of Hoechst-stained nuclei located within multinucleated cells, positive to sarcomeric myosin (Figure 2(b)). The altered differentiated muscle phenotype was also confirmed by western blot analysis, revealing a drastic downmodulation of the sarcomeric myosin heavy chain expression, a specific marker of myogenic differentiation (Figure 2(c)). Of note, C2C12 cells stably transfected with wild type SOD1 cassette (MLC/SOD1^Wt^) did not show any morphological differences compared to control C2C12 cell lines (data not shown).

**Figure 3:**
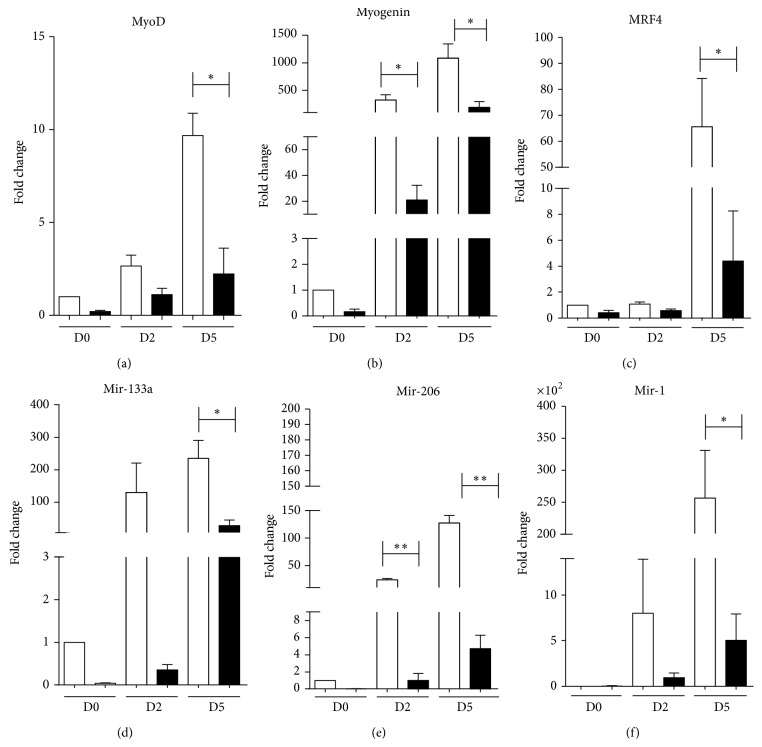
Mutant SOD1 gene downmodulates the players involved in C2C12 differentiation. Real time PCR for MyoD (a), Myogenin (b), MRF4 (c), miR133a (d), miR206 (e), and miR1 (f) in both control (white bars) and transfected cells (black bars). D0, D2, and D5 referred to days of culture in differentiation condition. Data are represented as mean ± SEM (^*^ *P* < 0.05; ^**^ *P* < 0.005).

A key myogenic factor that triggers myoblast differentiation is MyoD [12, 13], which resulted in significant downregulation throughout the time course of differentiation in C2C12 MLC/SOD1^G93A^ cells (Figure 3(a)). Myogenin is the myogenic factor that functions downstream of MyoD and plays a critical role in triggering terminal differentiation process of myoblasts [12, 14]. Myogenin expression resulted in significant downregulation in C2C12 MLC/SOD1^G93A^ cells during differentiation (Figure 3(b)). The final stage of skeletal muscle differentiation and maturation program is also dependent on the concerted action of another myogenic factors, namely, MRF4, which promotes the activation of myosin heavy chain expression [12]. Real time PCR analysis revealed a significant reduction of MRF4 transcripts in C2C12 MLC/SOD1^G93A^ cells during differentiation, compared to control C2C12 myotubes (Figure 3(c)).

Recent works have shown that among genes which are important for proper muscle differentiation and function, microRNAs (miRNAs) play a crucial role [15–17]. Among them miR133, miR206, and miR1 are abundantly expressed in muscle tissue and specifically induced during myogenesis and C2C12 differentiation [18]. It has been reported that miR1 and miR133 are involved in a complex molecular mechanism by which miR1 induces the downmodulation of an inhibitor of muscle differentiation, namely, histone deacetylase (HDAC) 4 [19] and miR133, which is clustered on the same chromosomal loci of miR1 and enhances myoblast proliferation inhibiting the serum response factor (SRF). In addition, miR206 facilitates satellite cell differentiation [20, 21] by restricting its proliferative potential through the repression of Pax-7 expression [22]. These findings implicate these myomiRNAs in a complex regulatory loop to control cell proliferation, commitment, and differentiation. Real time PCR analysis revealed a significant downmodulation of miR133, miR206, and miR1 during the differentiation time course of C2C12 MLC/SOD1^G93A^ cells compared to control C2C12 cells (Figures 3(d), 3(e), and 3(f)).

### 3.2. Muscle Specific Expression of Mutant SOD1 Gene Perturbs Signaling Pathways of Muscle Differentiation

The activation of a specific developmental program requires the integration of multiple extrinsic signals from the cell membrane that culminate in changes of nuclear gene expression patterns. Among the known signal transduction intermediates in muscle cells, the serine/threonine kinase AKT and the mitogen-activated protein kinases (MAPK) have been shown to modulate myogenic differentiation [23]. In a previous study [6], we have demonstrated that muscle specific expression of SOD1^G93A^ gene in transgenic animals promotes a reduction of the phosphatidylinositol 3-kinase (PI3K)/Akt pathway and leads to muscle atrophy.

**Figure 4:**
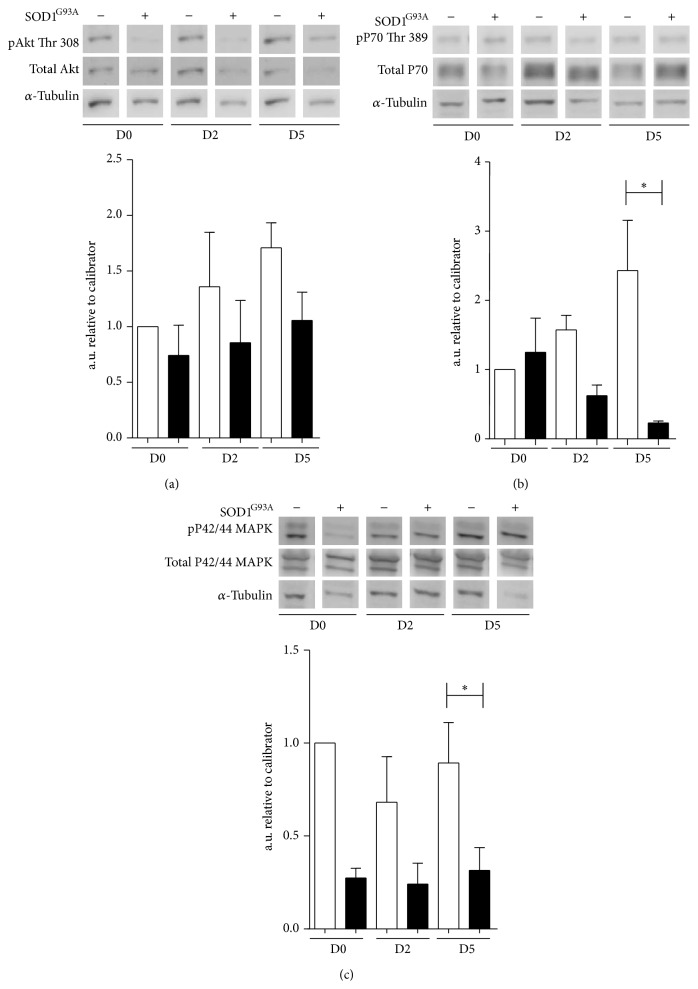
Muscle specific expression of human mutant SOD1 gene alters signaling pathways in muscle differentiation. (a) Representative western blot analysis for phospho-Akt (Thr 308) and total Akt expression (upper panel) and densitometric analysis (lower panel) of the ratio between phosphorylated Akt and the total form (lower panel) in C2C12 (white bars) and C2C12 MLC/SOD1^G93A^ (black bars). (b) Representative western blot analysis for phospho-P70 (Thr 389) and total P70 expression (upper panel) and densitometric analysis (lower panel) of the ratio between phospho-P70 and total P70 (lower panel) in C2C12 (white bars) and C2C12 MLC/SOD1^G93A^ (black bars). (c) Representative western blot analysis of phospho-p42/44 MAPK and p42/44 MAPK expression (upper panel) and densitometric analysis (lower panel) of the ratio between phospho-p42-44 MAPK and total p42/44 MAPK in C2C12 (white bars) and C2C12 MLC/SOD1^G93A^ (black bars). D0, D2, and D5 referred to days of culture in differentiation condition. Data are represented as mean ± SEM (^*^ *P* < 0.05).

In this study, we explored whether the inhibitory effects of mutant SOD1 gene on myogenic program perturb the relevant signaling pathways of the myogenic program.  Figure 4 shows that the absolute ratio of pAkt/Akt was negatively regulated (Figure 4(a)) and pP70/P70, the downstream effector of AKT, was significantly reduced in C2C12 MLC/SOD1^G93A^ cultures compared with control C2C12 cells (Figure 4(b)). In addition we observed a significant downmodulation of the phosphorylated active form of a factor associated with MAPK differentiation cascade, namely, ERK1/2 (Figure 4(c)).

Overall these results demonstrate that the postmitotic expression of SOD1 mutant gene prevents C2C12 differentiation, affecting the activation of the muscle regulatory factors, muscle miRNA, and the signal transduction cascades responsible for myogenic differentiation, and might impinge the maintenance of the muscle phenotype.

### 3.3. Muscle Specific Expression of SOD1^G93A^ Impinges the Identity of Muscle Cells and Promotes a FAPs Phenotype in C2C12 Myogenic Cells

**Figure 5:**
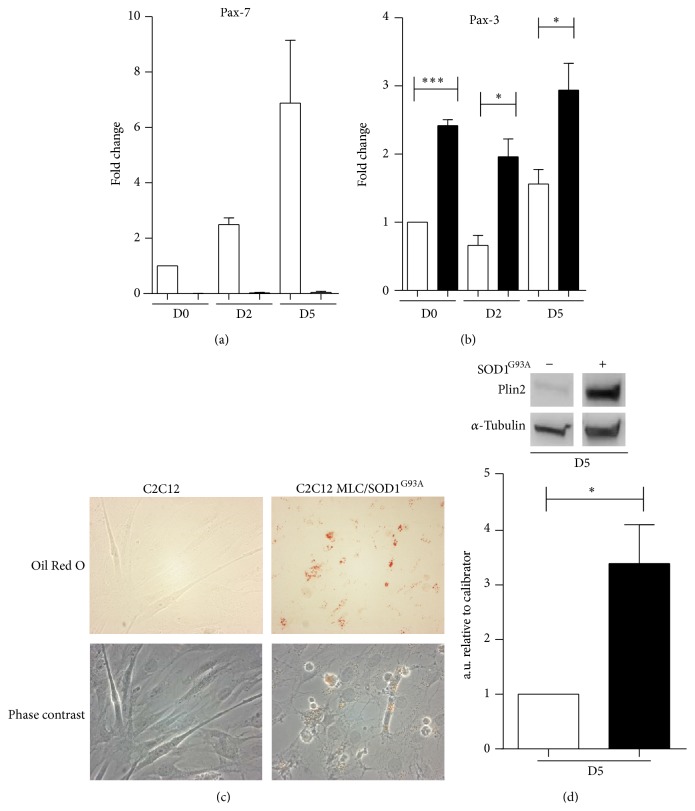
C2C12 MLC/SOD1^G93A^ cells exhibit adipogenic features. Real time PCR analysis for Pax7 (a) and Pax3 (b). (c) Upper panel shows Oil Red O staining and phase contrast of control C2C12 and C2C12 MLC/SOD1^G93A^ transfected cells at day 5 in culture. (d) Upper panel shows representative western blot analysis of Plin2 and relative densitometric analysis (lower panel) of the ratio between Plin2 and *α*-tubulin in C2C12 (white bars) and C2C12 MLC/SOD1^G93A^ (black bars). D0, D2, and D5 referred to days of culture in differentiation condition. Data are represented as mean ± SEM (^*^ *P* < 0.05; ^***^ *P* < 0.0005).

To validate this hypothesis, we analyzed the expression of Pax-7, which is a key factor that triggers the specification of uncommitted skeletal muscle progenitors to myogenic cells [24]. Of note, we observed a complete inhibition of Pax-7 expression in C2C12 MLC/SOD1^G93A^ cells compared to C2C12 myoblasts, suggesting that SOD1^G93A^ expression not only inhibits muscle differentiation but also confers to transfected cells an immature state (Figure 5(a)).

Recently, it has been demonstrated that satellite cells in adult muscle are bipotential stem cells that can give rise to brown adipogenic as well as myogenic progenitors [25]. The lineage switch between myogenic and brown adipogenic commitment is controlled by the muscle specific miR133a, which is highly expressed in satellite cells and can repress the expression of adipogenic markers to enforce myogenic commitment in satellite cells [25].

Further, recent evidences revealed that Pax-3 transcription factor, whose ectopic expression in C2C12 myoblasts efficiently inhibits myogenic specification [26], plays a pivotal role during differentiation into adipocytes cells of human-induced pluripotent stem cells [27]. In addition, the exclusive expression of Pax-3 or MyoD gene in stem cells allows a clear and distinct choice between myogenic and fibro/adipogenic potential cell lineage [28]. Based on these evidences, we performed real time PCR and histochemical analysis in both C2C12 and C2C12 MLC/SOD1^G93A^ cells to evaluate the potential adipogenic features in C2C12 MLC/SOD1^G93A^ transfected cell lines. As shown in Figure 5(b) the levels of Pax-3 transcript were significantly upregulated in C2C12 MLC/SOD1^G93A^ during all stages in culture, compared to C2C12. Moreover Oil Red O staining revealed the accumulation of intracellular lipid droplets in C2C12 MLC/SOD1^G93A^ cells (Figure 5(c)). These data were corroborated by western blot analysis for Perilipin 2 (Plin2), a marker of fatty acids uptake and storage [29]. Western blot analysis revealed that Plin2 protein was accumulated in C2C12 MLC/SOD1^G93A^ during the differentiation process compared to control cell line (Figure 5(d)). All these results together with the data of miR133a and MRFs downmodulation in C2C12 MLC/SOD1^G93A^ (Figure 3) suggest that muscle specific expression of SOD1 mutant gene inhibits myoblasts differentiation and promotes adipogenic features in C2C12 cells through a miR133a and Pax-3 dependent mechanism.

Fibro-adipogenic progenitors (FAPs) are multipotent mesenchymal cells residing in skeletal muscle interstitium [30–32]. These cells are negative for CD31, CD45, and *α*7 integrin surface antigens and are characterized by the expression of the stem cell antigen 1 (Sca1) [30]. FAPs convert environmental cues into signals that modulate muscle regeneration or turn themselves into fibro-adipocytes, inducing fat deposition and fibrosis under pathologic conditions, such as dystrophic muscles [33].

**Figure 6:**
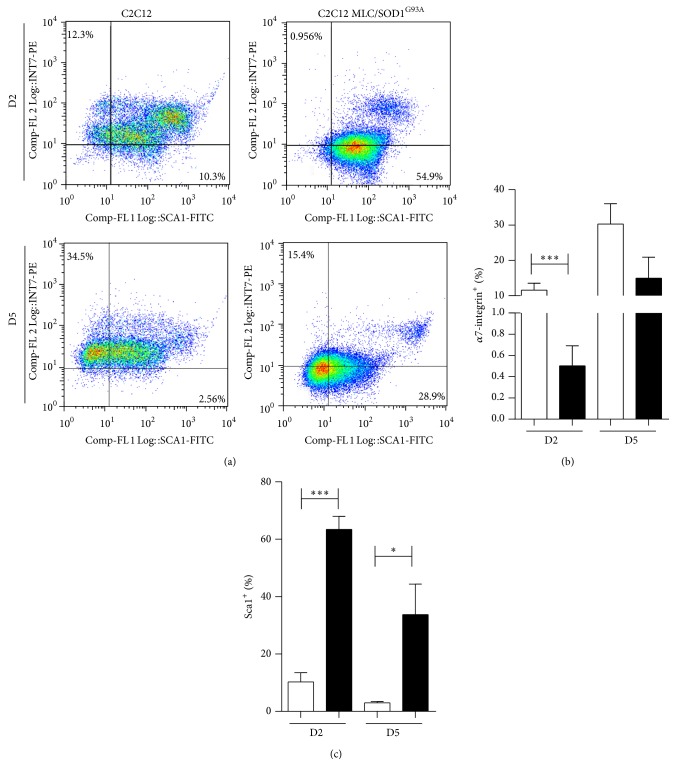
Expression of mutant SOD1 promotes a FAPS phenotype in C2C12 cells. (a) Flow cytometry profile for *α*7 integrin and Sca1 expression from control C2C12 and transfected C2C12 MLC/SOD1^G93A^ cells at days 2 and 5 in differentiation medium. (b, c) Histograms of the percentage of *α*7 integrin (b) and Sca1 (c) positive cells of C2C12 (white bars) and C2C12 MLC/SOD1^G93A^ (black bars) cells. D2 and D5 referred to days of culture in differentiation condition. Data are represented as mean ± SEM (^*^ *P* < 0.05; ^***^ *P* < 0.0005).

To verify whether postmitotic expression of SOD1 mutant gene promotes a FAPs phenotype in C2C12 cells, we performed FACS analysis (Figure 6(a)) for Sca1, CD31, CD45, and *α*7 integrin. C2C12 and C2C12 MLC/SOD1^G93A^ cells were negative for CD31 and CD45 antigens (data not shown). C2C12 were mainly *α*7 integrin^+^ and a low percentage of them was Sca1^+^ and *α*7 integrin^−^ (Figures 6(a)–6(c)). In contrast, C2C12 MLC/SOD1^G93A^ were mainly Sca1^+^ and *α*7 integrin^−^, with a significant reduction in the number of *α*7 integrin^+^ cells (Figures 6(a)–6(c)). These results clearly evidenced the presence of fibro/adipogenic features in C2C12 MLC/SOD1^G93A^. The molecular mechanism that, in concert with environmental cues, controls the identity and activity of FAP cells involves a HDAC-regulated network; this network consists of muscle specific miRNAs that target two alternative variants of the SWI/SNF chromatin remodeling complex, BAF60A and BAF60B, and favor the formation of a BAF60C-based SWI/SNF complex able to confer on MYOD the ability to activate the myogenic program [34].

**Figure 7:**
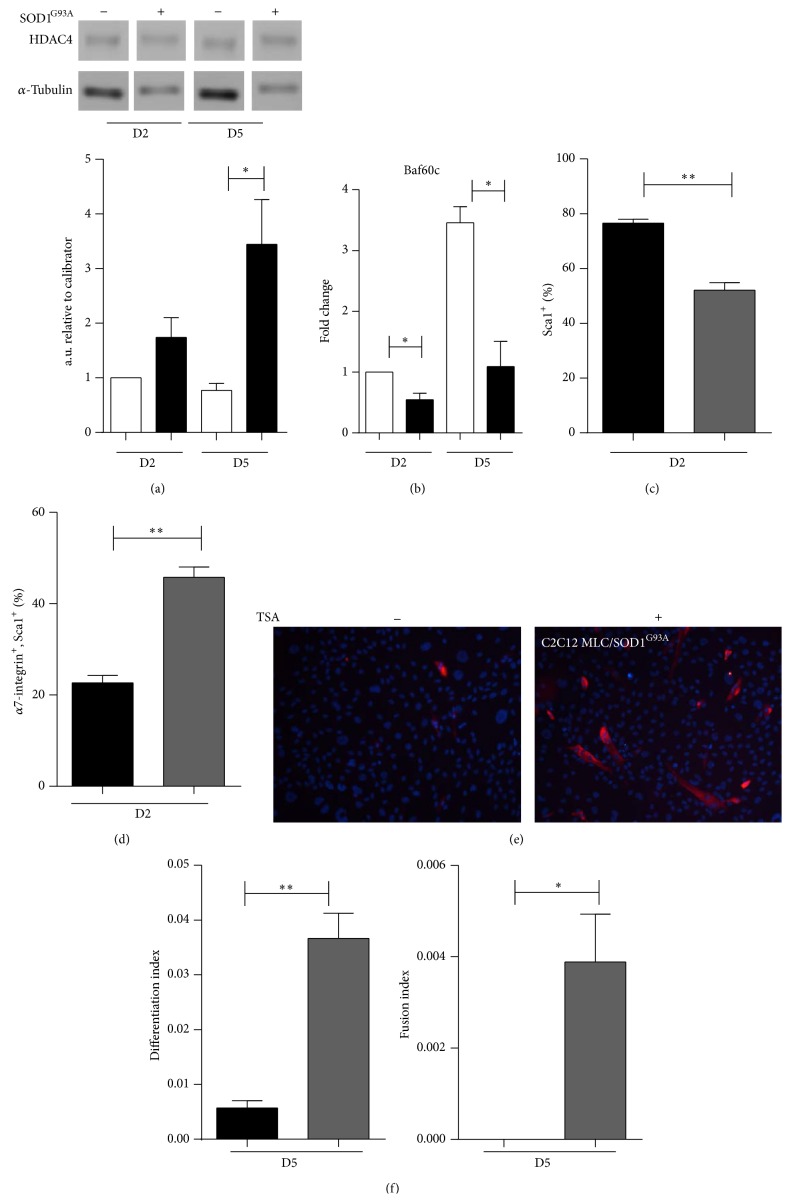
The acquisition of fibro-adipogenic features involves a HDAC-regulated network. (a) Upper panel shows western blot analysis of HDAC4 expression in C2C12 and C2C12 MLC/SOD1^G93A^ cells and densitometric analysis (lower panel) of the ratio between HDAC4 and *α*-tubulin. (b) Real time PCR for BAF60C in C2C12 (white bars) and C2C12 MLC/SOD1^G93A^ (black bars). (c, d) Histograms of the percentage of Sca1 (c) and positive *α*7 integrin cells (d) of control C2C12 MLC/SOD1^G93A^ (black bars) cells and TSA treated C2C12 MLC/SOD1^G93A^ cells (grey bars). D2 referred to days of culture in differentiation condition. (e) Representative immunofluorescence analysis for MHC after 5 days in differentiation medium in untreated (left panel) and TSA treated (right panel) C2C12 MLC/SOD1^G93A^ cells. (f) Histograms of the differentiation index (left panel) and fusion index (right panel) in control (black bars) and treated cells (grey bars). D2 and D5 referred to days of culture in differentiation condition. Data are represented as mean ± SEM (^*^ *P* < 0.05; ^**^ *P* < 0.005).

We analyzed the protein and transcript levels, respectively, of HDAC4 and BAF60C in both C2C12 and C2C12 MLC/SOD1^G93A^ cells. We observed that HDAC4 was significantly upregulated in cultures of C2C12 MLC/SOD1^G93A^ cells compared to control differentiated (day 5 in DM) C2C12 cells (Figure 7(a)). In contrast, BAF60C was significantly downregulated in C2C12 MLC/SOD1^G93A^ cells (Figure 7(b)).

To better correlate the induction of FAPs phenotype and HDAC activity with SOD1^G93A^ expression and toxic properties, we treated C2C12 MLC/SOD1^G93A^ cells with the HDAC class II inhibitor Trichostatin (TSA) and analyzed the signature profile of both FAPs and myogenic cells. Cytofluorimetric profile revealed that TSA treatment induced a significant downmodulation of Sca1^+^ and *α*7 integrin^−^ cells (Figure 7(c)) and an increased percentage of double positive Sca1^+^ and *α*7 integrin^+^ cells (Figure 7(d)). Of note we did not observe any significant difference in the number of *α*7 integrin^+^ Sca1^−^ cells after treatment (data not shown) suggesting that TSA treatment is able to counteract the establishment of a FAP phenotype but it only partially rescued the muscle phenotype. To support this evidence, we induced muscle differentiation in C2C12 MLC/SOD1^G93A^ cells treated with TSA and we revealed that inhibition of HDAC activity partially rescued myogenic differentiation, promoting myoblast fusion and differentiation (Figures 7(e)-7(f)).

Overall these data suggest that the postmitotic expression SOD1^G93A^ mutant gene promotes a FAPS phenotype in C2C12 cells, by upregulating HDAC4 protein and preventing the BAF60C-SWI/SNF complex myogenic commitment.

## 4. Discussion

In this work we defined the specific toxic effects of postmitotic expression of mutant SOD1^G93A^ gene on the myogenic program, demonstrating that mutant SOD1^G93A^ alters the identity of muscle cells, affects cell homeostasis, and inhibits muscle differentiation.

The physiological activity of the metalloenzyme SOD1 is to detoxify the cells from the accumulation of free radicals, converting superoxide, a toxic by-product of mitochondrial oxidative phosphorylation, to water or hydrogen peroxide. In contrast, the mutant SOD1 possesses a toxic property that is responsible for the pathogenic mechanism of ALS, a neurodegenerative disease associated with the degeneration of motor neurons, muscle atrophy, and paralysis.

Different studies also support the evidence that skeletal muscle is a primary target of mutant SOD1 toxicity in mice [5, 35], indicating that dysfunctions of affected muscle cells are not only a marginal consequence of denervation associated with motor neurons loss, but a direct consequence of cell muscle toxicity of mutant SOD1 [36]. The understanding of the mechanisms involved in mutant SOD1 toxicity in muscle may facilitate the design of treatments directed toward this specific tissue to treat ALS or at least to delay disease progression.

The aim of our work was to define the responses of myogenic cells to the toxic effects of SOD1^G93A^ and the signaling pathways that mediated the toxic properties of mutant SOD1 product. To this purpose, we generated a stable transfected C2C12 cell line, overexpressing the mutated isoform of the SOD1 gene under the control of the MLC promoter [11, 37].

Postmitotic expression of SOD1^G93A^ gene induced an excess of oxidative stress, as evidenced by the increased expression of gp91 protein, and impaired muscle differentiation and fusion of C2C12 cells, inducing a significant downregulation in the expression of molecular markers of myogenic differentiation, like MyoD, Myogenin, MRF4, and sarcomeric myosin heavy chain.

Epigenetic factors, including miRNAs expression, play important role in muscle homeostasis and represent good molecular markers to define the stage of myogenic program and to monitor the effects of toxic factors on the modulation of muscle phenotype. miR206, miR133a, and miR1 [18, 20, 38] contribute to the proper development of the myogenic program, and their alteration can impair the myogenic differentiation. Based on this evidence, we investigated their expression levels in C2C12 MLC/SOD1^G93A^ cells and demonstrated that transfected MLC/SOD1^G93A^ induced a significant downmodulation of all muscle microRNAs analyzed.

The myogenic program is governed by specific pathways of signal transduction. Here we have demonstrated that the inhibitory effect of the toxic SOD1^G93A^ protein on myogenic differentiation acts negatively on two major signaling pathways involved in muscle differentiation, such as the AKT/p70 and MAPK pathways.

These results suggest that muscle expression of SOD1^G93A^ impinges muscle differentiation and might alter the identity of muscle cells in line with previous studies that demonstrate the impairment of myofiber-associated skeletal muscle satellite cells function in SOD1-G93A mice [39] and the altered expression of myogenic regulatory factors in the mouse model of amyotrophic lateral sclerosis [40].

Recent evidences indicate that ROS levels correlate with a high concentration of glucose. Aguiari and colleagues [9] have shown that high glucose growth medium induces an increase in ROS production and promotes the adipocyte differentiation of muscle derived stem cells [9].

Since C2C12 MLC/SOD1^G93A^ cells show higher levels of oxidative stress and impaired myogenic differentiation process, modulating relevant genes and myomiRNA of the maintenance of the muscle phenotype, we supposed a link between toxic properties of SOD1^G93A^ and activation of adipogenic differentiation. In particular, we demonstrated that transfected MLC/SOD1^G93A^ cells show low levels of miR133a, Pax-7, and MyoD expression and high levels of Pax-3 and Perilipin 2, a marker of lipid droplets.

Our data support the evidences by Yin and colleagues [25] who showed that miR133a controls satellite cells commitment to the adipocyte lineage; in this work the authors demonstrated that miR133a inhibition promotes proadipogenic differentiation of satellite cells and that the new-formed preadipocytes completely lose the expression of both Pax-7 and MyoD [41]. Moreover, Mohsen-Kanson et al. [27] demonstrated that Pax-3 plays a pivotal role during stem cell differentiation into adipocytes and that the exclusive expression of Pax-3 or MyoD allows cells to choose between myogenic and fibro/adipogenic cell lineage.

Our data indicate that muscle expression of SOD1^G93A^ induced deregulation of myogenic process, led to the impairment of myoblast differentiation, and promoted adipogenic commitment.

The fibro-adipogenic precursors or FAPs cells are bipotent cells positive for Sca1 and negative for *α*7 integrin, CD31, and CD45, markers of satellite cells, endothelial cells, and hematopoietic cells, respectively. FAPs are mesenchymal cells residing in skeletal muscle interstitium and quiescent in physiological conditions and are efficiently activated to proliferate after muscle injury [30]. FAPs convert environmental factors into specific signals that can modulate muscle regeneration [34]. It has been shown that FAPs may exacerbate the dystrophic phenotype turning into fibro-adipocytes, mediating fat deposition and fibrosis [33] and thereby disrupting the environment conducive for muscle regeneration.

The trigger that controls FAP lineage commitment and activity is currently unknown.

Interestingly, it has been demonstrated that, during ischemia-induced regeneration, oxidative stress negatively modulates myogenic differentiation [42] and it is well known that oxidative stress is a hallmark of chronic disease including the Duchenne muscle dystrophy where FAPs have been deeply studied.

Here we demonstrate, by histochemical assay and FACS analysis, the presence of lipid droplets in C2C12 MLC/SOD1^G93A^ cells and their significant and exclusive positivity for Sca1, demonstrating that C2C12 MLC/SOD1^G93A^ cells share common features with FAPs.

A recent study has revealed a link between HDACs, myomiRs, and chromatin remodeling that underlies FAPs commitment to the “promyogenic” phenotype [34]. The authors demonstrated that the signaling pathway that mediates the transition from myogenic to fibro/adipogenic phenotype requires the action of miR1, miR206, and miR133a, which favor the incorporation of specific core-protein, BAF60C, into the chromatin remodeling SWI/SNF complex. Moreover, the authors also demonstrated that in a murine model of Duchenne dystrophy treatments with the HDACs inhibitor (TSA) address FAPs to a proper myogenic fate, blocking the fibro-adipogenic one [34].

Since HDAC4 regulates FAPs fate and since our transfected cell line shares common features with FAPs, including higher levels of HDAC4 protein, lower levels of BAF60c, and deregulation of myomiRNA expression, we hypothesized that HDAC4 is responsible for C2C12 MLC/SOD1^G93A^ adipogenic choice. To prove our hypothesis we treated C2C12 MLC/SOD1^G93A^ cells with TSA and interestingly we observed a partial rescue of the myogenic phenotype. This suggests that inhibition of HDAC activity interferes with the establishment of a FAP phenotype and partially rescues muscle differentiation.

## 5. Conclusions

Three main conclusions can be drawn from the present work; the postmitotic expression of SOD1 mutant gene (1) induces the impairment of the myogenic differentiation process of C2C12 cells; (2) triggers the myoblast towards an adipogenic phenotype; (3) promotes FAPs features in C2C12 cells by epigenetic changes that involve HDACs proteins.

Further studies will clarify the different molecular mechanisms that are modulated by multiple toxic effects of mutant SOD1 protein in skeletal muscle and whether oxidative stress can represent a determinant for myoblasts choice toward a fibro/adipogenic fate.
